# *SF3a1*: A Novel Potential Tumor Biomarker or Therapeutic Target

**DOI:** 10.7150/jca.103209

**Published:** 2025-04-09

**Authors:** Xueqian Shuai, Yaoqi Sun, Shupeng Liu, Zhongping Cheng

**Affiliations:** 1 Department of Obstetrics and Gynecology, Shanghai Tenth People's Hospital, Tongji University School of Medicine, Shanghai, 200040, China.; 2 Shanghai Fourth People's Hospital, Tongji University School of Medicine, Shanghai, 200434, China.; 3 Gynecological Minimally Invasive Surgery Institute, Tongji University School of Medicine, Shanghai, 200331, China.

**Keywords:** alternative splicing, prognostic biomarker, SF3a1, therapeutic target, tumorigenesis.

## Abstract

Alternative splicing is an evolutionarily conserved and essential cellular process that is catalyzed by a multi-complex spliceosome. Dysregulation of this process has been implicated in various tumors over the recent years. *SF3a1* is a critical subunit of U2 small nuclear ribonucleoprotein (snRNP) in the spliceosome, which has been found to be aberrant in several human diseases. Recent reports suggest that *SF3a1* might be a novel therapeutic target. However, a comprehensive description of *SF3a1* is lacking. In this review, we present the findings of *SF3a1* from protein structure, biological function to strong associations with human diseases including cancer. Studies have reported that *SF3a1* dysregulation and associated alternative splicing events mediate tumorigenesis and other immune-related disorders. However, further functional and mechanistic studies are needed to fully understand the regulatory network of *SF3a1* in human diseases. In conclusion, *SF3a1* could serve as a promising prognostic biomarker and therapeutic target for specific cancer types, including prostate cancer, colorectal cancer and hepatocellular carcinoma.

## Introduction

Pre-mRNA alternative splicing (AS) is an essential process that ensures proteome diversity from a limited number of protein-coding genes in eukaryotic genomes. This process is carried out by a dynamic ribonucleoprotein complex, also known as the spliceosome. Five snRNPs and over 100 non-snRNP proteins assemble sequentially to form the spliceosome during the process 1, 2. Abnormal status of these proteins cause aberrant AS and lead to disease including tumor 3. U2 snRNP is one of the snRNPs that assembles the E and A complexes at the early stage of AS 4. It recognizes branchpoint sequence (BPS) of the intron through SF3a and SF3b complexes 5, 6. Binding of SF3a and SF3b complexes to a 20-nucleotide region upstream of the BPS anchors U2 snRNP to pre-mRNA and formed A complex in an ATP-dependent manner 4-6. As one of the main components of U2 snRNP, the SF3a complex contains three subunits: *SF3a1*/*SF3a1*20, SF3a2/SF3a66 and SF3a3/SF3a60 7, 8. Each subunit of SF3a is essential for human cell viability and pre-mRNA splicing 9, 10. Aberrant expression of these proteins has been found in several malignant tumors, suggesting their involvement in tumor development and progression 11-13.

Given the key functions of *SF3a1* in spliceosome and the extensive regulatory effects of other U2 snRNP components on tumor biology 12, we present a comprehensive overview of *SF3a1's* structure and biological function, particularly in the context of tumorigenesis. The correlation between *SF3a1* and cancers, as well as other immune-related diseases, including autoimmune disorders and infections, suggests that it could serve as a promising prognostic marker and therapeutic target. However, there remains a significant gap in functional and mechanistic research that needs to be addressed.

## The structure of *SF3a1* and its role in spliceosome

### The structure of the *SF3a1* gene and protein

**Figure 1:**
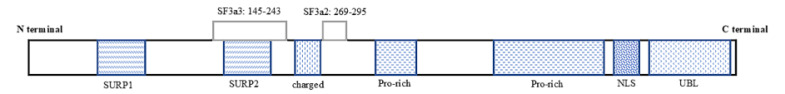
** Schematic representation of *SF3a1* protein.** The numeric range referred to the amino acids spanning on *SF3a1* that interacted with SF3a2 or SF3a3. SURP, suppressor-of-white-apricot and prp21/spp91; NLS, nuclear localization signal; UBL, ubiquitin-like.

Human *SF3a1* gene is located on chromosome 22q12.2, which containing 16 exons and 15 introns 14. It is transcribed into three transcript variants with 3.2, 3.8 and 5.7 kilobases respectively, and produces a 793-amino acid protein rich in prolines and charged amino acids. *SF3a1* has been identified to be homologous to splicing factor PRP21p in *Saccharomyces cerevisiae* 15*.* The N-terminal of *SF3a1* contains two suppressor-of-white-apricot and prp21/spp91 (SURP) domains, and the C-terminal contains a nuclear localization signal (NLS) and a ubiquitin-like (UBL) domain 15, 16 (Fig. 1). The SURP2 domain mediates the interaction of *SF3a1* with SF3a3, while the 130-amino acid region between SURP2 and Pro-rich domains mediates the interaction of *SF3a1* with SF3a2 15. Nuclear magnetic resonance (NMR) spectroscopy revealed that Leu169 in SURP2 domain of *SF3a1* was indispensable for the SF3a complex formation (Fig. 1). It was proposed that *SF3a1* functioned as the scaffold and nuclear import determinant of SF3a complex, because no direct interaction between SF3a2 and SF3a3 was found 10, 17, 18.

### The interplay between *SF3a1* and various molecules

As one of the core components of spliceosome, *SF3a1* participates in multiple physiological activities by interacting with various molecules including proteins and RNAs.

**Figure 2:**
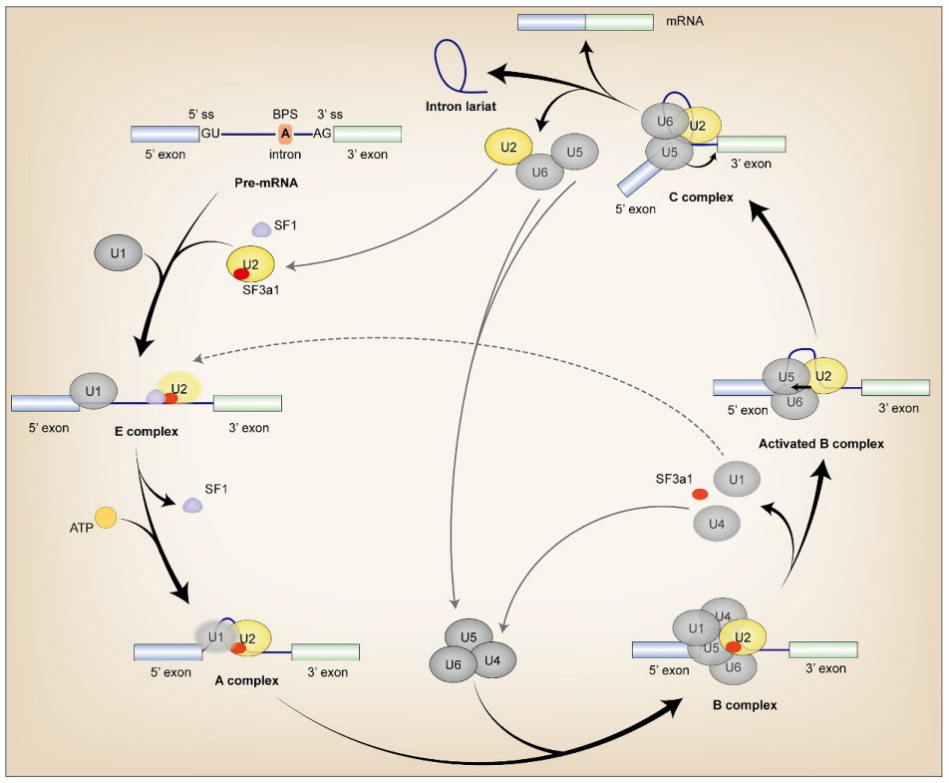
** The incorporation of *SF3a1* in spliceosome assembly.** Firstly, SF1 interacted with *SF3a1* to recruit U2 snRNP during complex E assembly, and *SF3a1* was necessary for anchoring U2 snRNP to pre-mRNA. Then, the interaction between *SF3a1* and U1 snRNA facilitated intron lariat formation in an ATP-dependent manner in complex A. Finally, before or during the first catalytic step of splicing, *SF3a1* was dissociated from the spliceosome. Except for the labels in the Figure, black arrow in the complex: transesterification; ss, splice site; gray lines referred to the splicing cycle; oval without black line edge indicated unstable binding.

During pre-mRNA AS, *SF3a1* interacts dynamically with various proteins and RNAs throughout spliceosome assembly. The SF3a heterotrimer is imported into the nucleus independently and targeted to Cajal bodies (CBs), where it is incorporated into the U2 snRNP 18-20. Although SF3a was found to be related to the 3' portion of the U2 snRNA and SF3b, the precise interaction between *SF3a1* and U2 snRNA has not yet been elucidated, although SF3a3 contacts both the bases of stem-loop I and a bulge in stem-loop III of U2 snRNA 21, 22. Then *SF3a1* binds to splicing factor 1 (SF1) via SURP1 domain to mediate recruitment of the U2 snRNP for efficient complex E assembly 1. During transition from the E to A complex, *SF3a1* interacts with the GCG/CGC RNA stem and the apical UUCG tetraloop of U1-SL4 in a sequence-specific manner. This interaction mediates the connection between U1 and U2 snRNPs, which identifies the 5' and 3' splice site of pre-mRNA respectively for functional spliceosome assembly and intron lariat formation. The RGG motif in the UBL domain at the carboxyl terminal of *SF3a1* plays a pivotal role in the interaction, while mutations at Y772 and Y773 in the UBL domain of *SF3a1* perturb the interplay 23-26. In the B complex, *SF3a1* binds to prp3 which is indispensable for U4/U6•U5 tri-snRNP formation 27 (Fig. 2).

In addition to spliceosome components, *SF3a1* also interacts with other proteins such as chromodomain helicase DNA-binding protein 1 (CHD1) to regulate pre-mRNA splicing. *SF3a1* bridged with CHD1 to enhance chromatin association and pre-mRNA splicing on specific genes by recognizing tri-methylation of histone H3 on lysine 4 (H3K4me3) 28. *SF3a1* activation facilitated proper splicing and accumulation of cohesion factor Sororin during S phase to maintain functional sister chromatid cohesion and subsequent cell cycle progression 29. *SF3a1* could also bind the N-terminal region of transcription factor SP1 with the function of this interaction remained unclear 30.

Thus, *SF3a1* exhibits complex interactions with various proteins and RNA motifs to ensure efficient and proper splicing of pre-mRNAs. Further exploration is needed to determine the precise role of *SF3a1* in the spliceosome and its upstream and downstream regulators.

## The role of *SF3a1* in tumors

Human tumors are characterized by splicing vulnerabilities 31. Like other core components of spliceosome, *SF3a1* has been identified as a risk factor in both hematologic and solid tumors (Table 1).

### The role of *SF3a1* in hematologic malignancies

Authoritative studies have shown *SF3a1's* involvement in hematologic malignancies. A whole-exome sequencing analysis revealed that *SF3a1* was mutated in myelodysplasia (MDS) patients with a predisposition to acute myeloid leukemia, which was further confirmed in a large series of myeloid neoplasms exhibiting features of MDS 38. Patients with blastic plasmacytoid dendritic cell neoplasm (BPDCN) were found to harbor somatic mutations in *SF3a1* and other RNA splicing components, however, *SF3a1* mutation status did not show statistically significant correlation with overall survival in this malignancy 32. In addition to tumorigenesis, *SF3a1* might also play a role in drug resistance. Recurrent *SF3a1* mutations were only present in non-responders to azacitidine (Aza) and erythropoietin (Epo) in transfusion-dependent, growth factor-resistant, low- and Int-1-risk MDS patients, although they were not able to predict therapeutic response 39. Dysregulated epigenetic modulation of *SF3a1* has also been found. DNA methylation analysis revealed that *SF3a1* was not downregulated by promoter hypermethylation in leukemia cells 40. The above studies demonstrated that *SF3a1* mutations occurred in specific types and subsets of hematologic malignancies. Although less frequently studied compared to SF3b1, *SF3a1* could be used as a complementary molecule to predict prognosis and treatment response in specific patients.

### The role of *SF3a1* in solid cancers

Research on *SF3a1* has been more abundant and in-depth in cancers compared to hematologic malignancies. A two-stage case-control study demonstrated a consistent association between two C alleles at rs2074733 in the *SF3a1* gene and an increased risk of pancreatic cancer. In addition, smoking or drinking cooperated with the harmful genotype of *SF3a1* to increase pancreatic cancer risk 33. *SF3a1* was also among the 12 mutated genes with novel but not penetrant non-synonymous SNPs in a family with gastric and rectal cancer 34. However, another hospital-based case-control study in a Chinese population indicated that four selected SNPs *(rs10376, rs5753073, rs2839998, and rs2074733)* of *SF3a1* were not significantly associated with colorectal cancer (CRC) risk, even after normalizing for smoking and alcohol use status 36. Limitations, including a small number of SNPs, samples, and environmental factors in the latter study, probably led to the contradictory results, which require further research for validation. Blood plasma cell-free DNA sequencing revealed major clonal mutations of *SF3a1* and others were found in all samples from one advanced CRC patient who showed rapid but not sustained response to chemotherapy, implying *SF3a1* might contribute to chemotherapy resistance and the pathogenesis of CRC 35. In a two-stage case-control study in China, TT alleles at rs5994293 in *SF3a1* gene also exhibited a significant correlation with higher hepatocellular carcinoma (HCC) risk, which additively interacted with smoking and alcohol consumption to increase HCC risk in HBsAg-negative participants in both the screened and combined cohorts 37. After applying multiple bioinformatic methods on the TCGA cohort, *SF3a1* was identified as one of the hub genes in the gene network of the top 800 OS-related AS events in HCC patients 41. Furthermore, *SF3a1* was among the 20 lactylation-related genes with prognostic grouping value in HCC. The low-risk group exhibited a more active immune response 42. In endometrial cancer, SF3a was among the up-regulated proteins in the human cell line Ishikawa after megestrol acetate treatment, implying a potential function of SF3a in predicting the response of gynecologic malignancy to hormonotherapy 43. In conclusion, *SF3a1* mutations are significantly correlated with the risk and prognosis of various cancers, particularly those of the digestive system, although some discordance exists, and further functional experiments are needed.

Several aberrantly expressed proteins in various cancers have been found to be regulated by *SF3a1*. Different isoforms of muscleblind-like 1 (MBNL1) were found to exert opposing functions in prostate cancer (PC). MBNL1 lacking exon 7, which tumor preferentially abandoned, led to DNA damage and subsequent inhibition of cell viability and migration 44. While the overall expression of MBNL1 was downregulated, exon 7 in the MBNL1 transcript was the most differentially included exon in several cancers including PC, and was essential for the homodimerization of the MBNL1 protein. *SF3a1* was necessary for the retention of exon 7 to exert pro-tumor effects 44. When PC3 cells were treated with novel and specific PolyPurine Reverse Hoogsteen (PPRH) hairpins for gene silencing against anti-apoptotic Survivin, *SF3a1* was identified among the important interactive gene nodes in the STRING network analysis and gene sets in GSEA of differentially expressed genes, indicating that SF3a1 participated in the apoptosis-related pathway of PC 45. The axis of *SF3a1*/ MBNL1 or Survivin isoforms might provide a promising subset of therapeutic targets and prognostic markers for PC. Furthermore, *SF3a1* was found to promote splicing landscape reprogramming and progression of metastatic castration-resistant PC through SOX6- GH22I030351 axis 46. And an integrative lactylome and proteome analysis based on liquid chromatography-tandem mass spectrometry (LC-MS/MS) revealed SF3A1 was lactylated, a novel type of post-translational modification, under both normoxia and hypoxia in oral squamous cell carcinoma (OSCC), which might contribute to the altered pre-mRNA splicing pattern and pathogenesis of OSCC 47.

Collectively, *SF3a1* presented as variant mutated forms in different hematologic and solid malignancies, and specific mutations were associated with patient outcomes and treatment responses, despite a few discordant findings, indicating *SF3a1* and related AS events could be potential and promising therapeutic targets. However, most studies have focused only on prognostic correlations, while functional and mechanistic research remains largely underexplored, particularly in terms of how mutation patterns of *SF3a1* influence U2 snRNP functionality, the subsequent mis-splicing of specific genes, and resulting tumorigenesis.

### The role of *SF3a1* in other immune-related diseases

*SF3a1* has also been implicated in non-tumor immune-related disorders, including autoimmune and infectious diseases. A systematic meta-analysis revealed that *SF3a1* SNPs were involved in genomic susceptibility repertoire of the most prevalent juvenile idiopathic arthritis subtype 48. Comprehensive functional genomic screens showed that SF3a promoted osteoarthritis progression by inducing collagen IIA expression 49. The expression level of SF3a, defined as an autoantigen gene of systemic lupus erythematosus (SLE), was upregulated in T lymphocytes of glomerulonephritis patients after immunosuppressive therapy 50. These findings indicated that *SF3a1* might act as an etiological factor and therapeutic target in specific auto-immune diseases.

SF3a was downregulated in THP-1 macrophage cells following mycobacterium tuberculosis (M. tb) H37Rv infection, implying the importance of SF3a in innate immune cells for pathogen defense 51. In addition to promoting inflammatory response, *SF3a1* also limited excessive and persistent inflammation by regulating the expression of mRNA isoforms in the toll-like receptor (TLR) signaling pathway. Negative regulators including sTLR4, Rab7b, and possibly IKKβb were upregulated through various AS events, while positive factors such as IRAK1, IKKβ, and CD14 were downregulated via intron retention regulated by *SF3a1*. MyD88 was another gene regulated by *SF3a1* via AS in the innate immune response 52, 53. Decreased expression of *SF3a1* by liver X receptor agonist T0901317 had been shown to inhibit inflammation via up-regulating the alternative splice short form of MyD88 mRNA 54.

These studies revealed that *SF3a1* dysregulation led to immune homeostasis disruption and the onset of immune-related disorders. Furthermore, *SF3a1* could serve as a target to prevent excessive inflammatory responses and the development of subsequent diseases.

## Conclusion

The idea that altered splicing underlay oncogenesis has gained attention and expanded the therapeutic scope of multiple tumors over the years. *SF3a1* appears to perform key functions within the SF3a complex in U2 snRNP through its dynamical interactions with various molecules. Currently, *SF3a1* mutations have been shown to be significantly correlated with hematologic and solid tumors. Several functional and mechanistic studies have elucidated the potential contribution of *SF3a1* to the pathogenesis of cancers including CRC, PC and HCC. Additionally, *SF3a1* also participates in autoimmune disorders and infectious diseases by regulating expression of isoforms of inflammatory factors. These findings suggest that *SF3a1* is involved in both tumorigenic and immune-related diseases, although further investigations are needed. It also suggests that *SF3a1* could be a novel therapeutic target for tumor and autoimmune disease treatment. The induction of immunogenic tumor-specific neoepitopes by SF3b1 mutations further implies its potential in tumor immunotherapy 31, 55.

Collectively, *SF3a1* is a component of the SF3a complex within the spliceosome, functions as a core regulatory factor in pre-mRNA AS, and plays pivotal roles in multiple biological processes, including tumor development. Aberrant expression of *SF3a1* and related AS events may serve as biomarkers or therapeutic targets for related diseases, especially cancers such as PC, CRC and HCC.

## Figures and Tables

**Table 1:** The clinical association between SF3a1 mutation and various hematologic and solid malignancies

Tumor type	Sample size^a^	SNP	Genotypes^b^	P value/ OR (95% CI)	Mutation frequency^c^	Clinical correlation	Ref.
BPDCN	50	N	N	N	0.02	no prognostic relation	32
Pancreatic cancer	298	rs2074733	(TT+CT)/CC	P=1.4E-02 /0.65(0.48-0.88)	0.60/0.70	lower risk	33
Pancreatic cancer	413	rs2074733	(TT+CT)/CC	P=3.0E-04 /0.54(0.41-0.73)	0.66/0.78	lower risk	33
Familial gastric and rectal cancer	8	N	T/C	N	0.88	possibly contributing to cancer	34
Familial gastric and rectal cancer	98	N	G/T	N	0.01	possibly contributing to cancer	34
CRC	1	rs1370165248	A/G	N	1	major clonal mutation	35
CRC	801	rs5753073	(AG+GG)/AA	P>0.05	0.25/0.28	no risk correlation	36
CRC	801	rs2839998	(GA+AA)/GG	P>0.05	0.55/0.53	no risk correlation	36
CRC	801	rs10376	(AC+AA)/CC	P>0.05	0.17/0.20	no risk correlation	36
CRC	801	rs2074733	(TC+CC)/TT	P>0.05	0.72/0.73	no risk correlation	36
HCC	378	rs5994293	(TG+GG)/TT	P=5.0E-04 /0.70(0.58-0.84)	0.65/0.49	higher risk	37
HCC	428	rs5994293	(TG+GG)/TT	P=5.0E-04 /0.70(0.58-0.84)	0.58/0.55	higher risk	37

N: not present. a: The size referred to patient number. b: The last genotype was used as the reference for OR calculations. c: The last number indicated mutation frequency of control group in case-control study. BPDCN, blastic plasmacytoid dendritic cell neoplasm; CRC, colorectal cancer; HCC, hepatocellular carcinoma; SNPs, single nucleotide polymorphisms.

## Abbreviations

snRNPs: small nuclear ribonucleoproteins
AS: alternative splicing
BPS: branchpoint sequence
SURP2: the second suppressor-of-white-apricot and prp21/spp91
NLS: nuclear localization signal
UBL: ubiquitin-like
NMR: nuclear magnetic resonance
CBs: Cajal bodies
SF1: splicing factor 1
CHD1: chromodomain helicase DNA binding protein 1
H3K4me3: histone H3 on lysine 4
MDS: myelodysplasia
BPDCN: blastic plasmacytoid dendritic cell neoplasm
Aza: azacitidine
Epo: erythropoietin
CRC: colorectal cancer
HCC: hepatocellular carcinoma
MBNL1: muscleblind-like 1
PC: prostate cancer
PPRH: PolyPurine Reverse Hoogsteen
LC-MS/MS: liquid chromatography-tandem mass spectrometry
OSCC: oral squamous cell carcinoma
SLE: systemic lupus erythematosus
M. tb: mycobacterium tuberculosis
TLR: toll-like receptor
